# Changes in aortic pulse wave velocity of four thoracic aortic stent grafts in an *ex vivo* porcine model

**DOI:** 10.1371/journal.pone.0186080

**Published:** 2017-10-05

**Authors:** Hector W. L. de Beaufort, Margherita Coda, Michele Conti, Theodorus M. J. van Bakel, Foeke J. H. Nauta, Ettore Lanzarone, Frans L. Moll, Joost A. van Herwaarden, Ferdinando Auricchio, Santi Trimarchi

**Affiliations:** 1 Thoracic Aortic Research Center, Policlinico San Donato IRCCS, University of Milan, San Donato Milanese, Italy; 2 Department of Civil Engineering and Architecture, University of Pavia, Pavia, Italy; 3 Institute of Applied Mathematics and Information Technologies (IMATI), National Research Council of Italy (CNR), Milan, Italy; 4 Department of Vascular Surgery, University Medical Center Utrecht, Utrecht, the Netherlands; Universita degli Studi di Roma La Sapienza, ITALY

## Abstract

**Objectives:**

Thoracic endovascular aortic repair (TEVAR) has been shown to lead to increased aortic stiffness. The aim of this study was to investigate the effect of stent graft type and stent graft length on aortic stiffness in a controlled, experimental setting.

**Methods:**

Twenty porcine thoracic aortas were connected to a pulsatile mock loop system. Intraluminal pressure was recorded at two sites in order to measure pulse wave velocity (PWV) for each aorta: before stent graft deployment (t_1_); after deployment of a 100-mm long stent graft (t_2_); and after distal extension through deployment of a second 100-mm long stent graft (t_3_). Four different types of stent grafts (Conformable Gore^®^ TAG^®^ Device, Bolton Relay^®^ Device, Cook Zenith Alpha^™^, and Medtronic Valiant^®^) were evaluated.

**Results:**

For the total cohort of 20 aortas, PWV increased by a mean 0.6 m/s or 8.9% of baseline PWV after deployment of a 100-mm proximal stent graft (P<0.001), and by a mean 1.4 m/s or 23.0% of baseline PWV after distal extension of the stent graft (P<0.001). Univariable regression analysis showed a significant correlation between aortic PWV and extent of stent graft coverage, (P<0.001), but no significant effect of baseline aortic length, baseline aortic PWV, or stent graft type on the percentual increase in PWV at t_2_ or at t_3_.

**Conclusions:**

In this experimental set-up, aortic stiffness increased significantly after stent graft deployment with each of the four types of stent graft, with the increase in aortic stiffness depending on the extent of stent graft coverage.

## Introduction

Thoracic endovascular aortic repair (TEVAR) is the first choice of treatment for patients with thoracic aortic diseases and suitable anatomy [1]. Understanding the effects of stent graft deployment on aortic physiology may help to explain some of the long-term outcomes after TEVAR. Stent graft deployment has been shown to significantly increase aortic stiffness [2], which has been clearly established as an independent predictor of cardiovascular mortality [3].

It is currently unknown which factors are related to the increase in aortic stiffness after TEVAR. The material of aortic stent grafts can be up to a 100 times stiffer than native aortic tissue [4, 5]. However, there are some differences in design among commercially available stent grafts. We hypothesized that different stent graft designs may have different stiffening effects. Moreover, we hypothesized that the length of aorta that is covered by stent graft is related to the increase in aortic stiffness after TEVAR. Therefore, the aim of this experimental study was to investigate whether the stiffening effects of stent graft deployment are dependent on stent graft type and length.

## Materials and methods

### Experimental set-up

The experimental set-up that was used has been described previously [6]. For the current study, twenty thoracic aortas of healthy pigs of a hybrid breed (10–12 months old, 160–180 kgs) were collected from a local slaughterhouse within 15 minutes after slaughter. The pigs were not raised or sacrificed for the purpose of this or any study but for commercial purposes. The aortas were transported and stored at 4°C in isotonic saline solution until used for experiments on the same day. At room temperature, excess connective tissue was removed and side branches were ligated from the aortic root to the celiac trunk. Under a continuous pressure of 96 mmHg [7], markers were placed on the outside aortic wall at the ascending aorta and at the level of the celiac trunk. Another marker was placed at the level where the aorta had a diameter of 23 mm, which marked the intended proximal stent graft position. The distance between ascending aorta and celiac trunk was measured using a wire and centimeter ruler.

### Pulse wave velocity measurement

**Fig 1:**
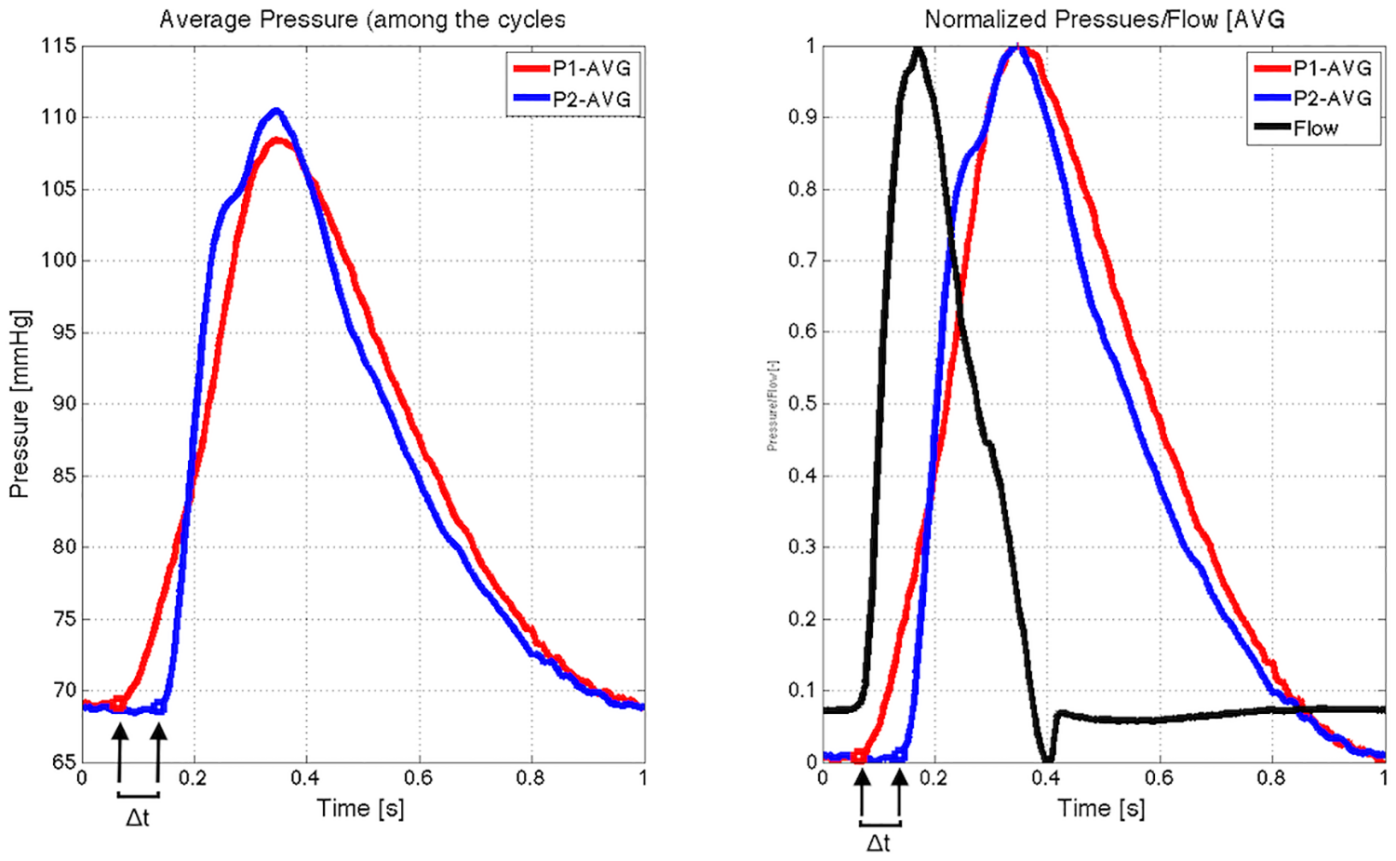
Generated pressure curves (average of five recorded cycles) in the ascending aorta and in the descending aorta at the level of the celiac trunk. **The distance between both pressure sensors was divided by the difference in time between the base of the pressure curves to calculate aortic pulse wave velocity**. Red: ascending aorta, blue: descending aorta.

The aortic specimens were placed within a 3D printed guide and connected to a pulsatile mock loop system [8], which is able to produce physiological pressures as a response to flow. The system was fixed at a heart rate of 60 beats per minute, output of 5L per minute, and peripheral resistance of 96 mmHg. Water at a temperature of 37°C was used for perfusion. The aortas were regularly with physiological saline solution to prevent tissue dehydration. Intraluminal pressure was recorded in the ascending aorta and at the level of the celiac trunk for five consecutive cardiac cycles using a needle connected to a pressure sensor (t_1_). The aorta was disconnected from the circuit and a 100-mm long stent graft with a diameter of 26 mm was deployed at the level where the aorta had a diameter of ±23 mm, to reach a 10% degree of oversizing. The aorta was then reconnected and intraluminal pressures were recorded for five cycles (t_2_). The stent graft was then extended with a second 100-mm long stent graft with a diameter of 22–24 mm and a 4 cm overlap, as is most commonly done in clinical practice. Intraluminal pressures were again recorded for five cycles (t_3_). Average aortic pulse wave velocity (PWV) at t_1_, t_2_, and t_3_ was calculated by dividing the distance between ascending aorta and celiac trunk by the average difference in time between the pressure peaks over the five recorded cycles (Fig 1).

**Fig 2:**
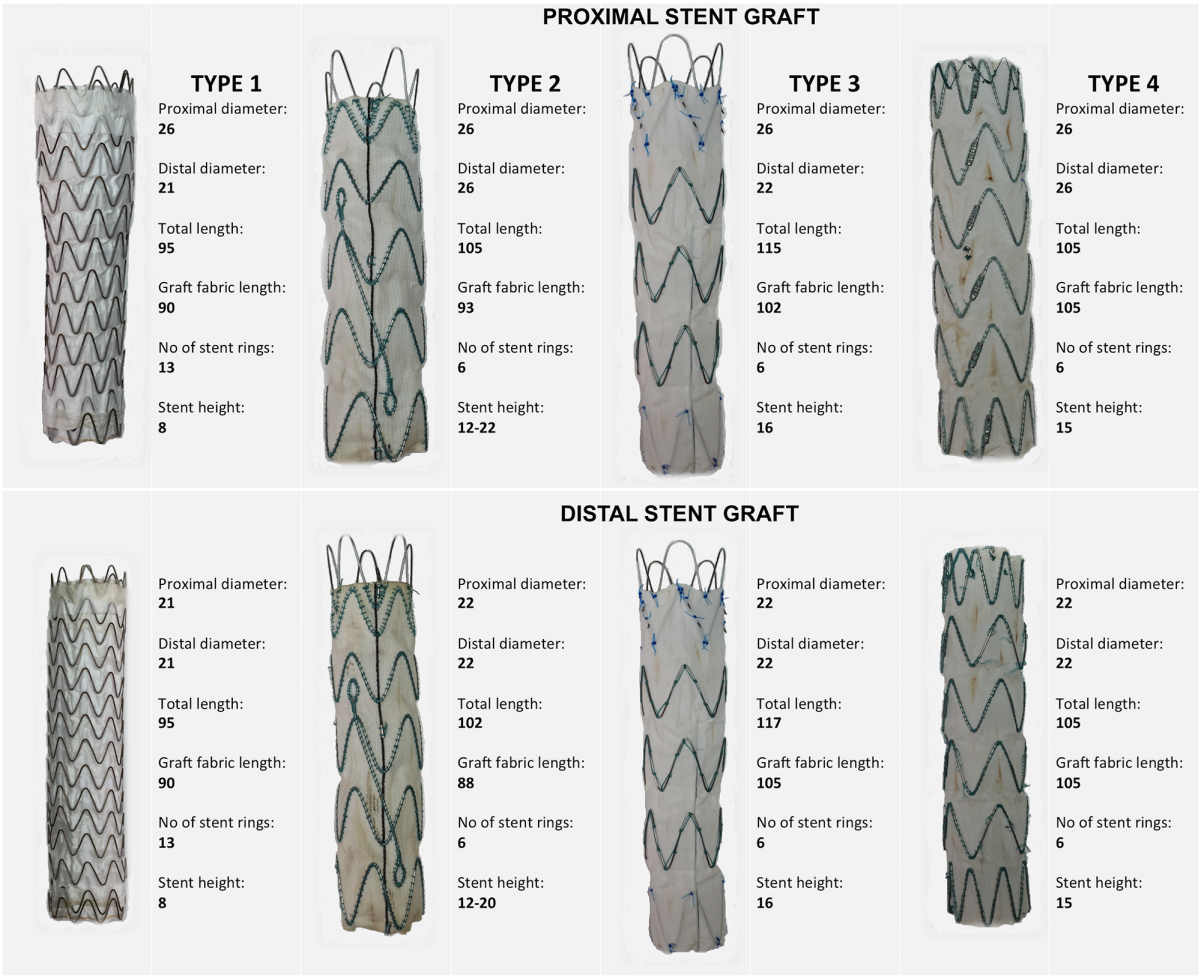
Description of the four types of stent grafts used for the experiments.

Four different stent graft types of comparable sizes were used; each stent graft type was tested in five different aortas. Stent graft type 1 (Conformable Gore^®^ TAG^®^ Device, W.L. Gore & Associates, Flagstaff, AZ, USA) is characterized by a continuous sinusoidal-shaped nitinol stent; Type 2 (Relay^®^, Bolton Medical, Barcelona, Spain) is made of Z-shaped nitinol stents with a woven polyester graft and one longitudinal nitinol bar. Type 3 (Zenith Alpha^™^, Cook Medical, Bloomington, IN, USA) is made of Z-shaped nitinol stents with a woven polyester graft and external fixation barbs. Type 4 (Valiant^®^, Medtronic, Santa Rosa, CA, USA) is made of Z-shaped nitinol stents with woven polyester graft. A description of the stent grafts used in the experiments is shown in Fig 2.

### Statistical analysis

IBM SPSS Statistics version 22.0 was used for statistical analysis. Normality of data was tested with the Shapiro-Wilk test. Univariable regression analysis was performed to test the effect of extent of stent graft coverage (t_1_ vs t_2_ vs t_3_) on aortic PWV, and the effect of baseline aortic length and PWV and stent graft type on the percentual increase in PWV at t_2_ and t_3_. Statistical significance was assumed at P<0.05.

## Results and discussion

Mean aortic length (n = 20) was 384.3 mm (± 28.44 mm) at baseline, and did not differ between the different stent graft groups (P = 0.294). Mean aortic PWV was 6.4 m/s (±0.7 m/s) at baseline, 7.0 m/s (±0.9 m/s) after deployment of a 100-mm proximal stent graft (t_2_), and 7.9 m/s (±1.5 m/s) after distal extension of the stent graft (t_3_). This corresponded to an 8.9% (±8.8%) increase in PWV at t_2_ (P<0.001) and a 23.0% (±17.4%) increase at t_3_ (P<0.001).

Baseline PWV increased by 8.0% (±5.4%) with stent graft type 1, by 4.7% (±8.8%) with stent graft type 2, by 8.4% (±8.3%) with stent graft type 3, and by 14.5% (±11.6%) with stent graft type 4 at t_2_. The differences in PWV increase at t_2_ did not reach statistical significance (P = 0.386). Baseline PWV increased by 21.3% (±12.2%) with stent graft type 1, 11.7% (±9.4%) with stent graft type 2, by 30.1% (±20.6%) with stent graft type 3, and by 27.9% (±23.3%) with stent graft type 4 at t_3_. The differences in PWV increase at t_3_ did not reach statistical significance (P = 0.462).

Univariable regression analysis showed a significant correlation between aortic PWV and extent of stent graft coverage, (P<0.001). There was no statistically significant effect of baseline aortic length or baseline aortic PWV on PWV increase at t_2_ (P = 0.666 and P = 0.527, respectively) or on the increase in PWV at t_3_ (P = 0.548 and P = 0.610). Full details on the data underlying these findings can be found as supporting information S1 Table.

The interaction between native aorta and aortic stent grafts is not fully understood and may depend on stent graft design [9, 10]. We used a controlled experimental set-up to test whether differences in stent graft design may account for different stiffening effects after stent graft deployment. This set-up eliminates variations in blood pressure, a known confounder for PWV measurements [11]. Thus, each stent graft type was tested under the same pressure and flow conditions. Our results show a net increase in aortic stiffness after stent graft deployment, dependent on stent graft length.

Increased aortic PWV is widely accepted as a predictor of cardiovascular mortality, because it is a marker of concomitant atherosclerotic disease. However, there are also independent pathophysiological consequences of increased aortic stiffness. A stiffer aorta has a reduced capacity for storage, causing the pulse wave to reach the resistance of the peripheral circulation at a higher velocity, increasing the amount of pulse wave reflections and cardiac afterload [12]. Furthermore, increased arterial stiffness is involved in the pathophysiology of hypertension, and TEVAR-induced aortic stiffness has been shown to lead to hypertension in young trauma patients [13]. The effects of the artificially induced aortic stiffening after TEVAR on cardiovascular remodelling thus merit further investigation [14].

Apparently, the most commonly used types of graft fabric and shapes of stent rings have at least similar stiffening effects. None of the currently used stent grafts have the capacity for longitudinal expansion. Developing a stent graft that does preserve the longitudinal distensibility of the native aorta while providing enough strength is a challenge that may be worth exploring. Aortic radial pulsatility may be preserved after stent graft deployment with the right amount of oversizing [15], thanks to the shape-memory of nitinol stents. However, the aorta is less stiff in longitudinal direction than in radial direction [16, 17], and a compliance mismatch between stented and non-stented aortic segments has been reported as an effect of stent graft deployment [9, 18]. It seems that the presence of a rigid stent graft leads to increased longitudinal strain and transmural pressure in proximal non-stented segments. Part of type Ia endoleaks and retrograde type A dissections occur at long-term and are attributed to progression of underlying aortic disease [19]. Since the length of stent graft was related to the increase in stiffness, one may speculate whether stent graft length is associated with complications.

Experimental set-ups have inherent limitations. No formal quality control of the porcine aortic specimens was performed, so the presence of connective tissue disorders or microscopic atherosclerosis could not be excluded, and may have confounded our results. Porcine tissue is more elastic than very aged [20] or aneurysmatic [21] human tissue, but might be similar to that of young individuals [22]. We used water, which has a lower viscosity than blood, for perfusion, as is also reported by other studies using ex vivo porcine aortas in a pulsatile flow circuit [23, 24]. The effect of blood viscosity on PWV is likely to be small in the high-speed environment of the aorta [25]. The aortas were slightly constrained by a 3D printed guide to approximate the movement of the thoracic aorta within the thoracic cavity. However, the absence of surrounding tissue may impact the mechanical properties of a vessel [26]. Finally, although the results of our experiments did not show major differences in stiffening effects of the studied stent graft types, our sample size was not sufficient to detect small differences that may have been confounded by small (<10%) differences in aortic length.

## Conclusions

Aortic stiffness increased significantly after stent graft deployment in this experimental study. The increase in aortic stiffness depended on the length of the aorta that was covered by stent graft but not on the type of stent graft.

## Supporting information

S1 Table Table with raw data on pulse wave velocity measurements per aortic specimen. (XLSX)
