# Correction: Changes in aortic pulse wave velocity of four thoracic aortic stent grafts in an *ex vivo* porcine model

**DOI:** 10.1371/journal.pone.0197453

**Published:** 2018-05-10

**Authors:** Hector W. L. de Beaufort, Margherita Coda, Michele Conti, Theodorus M. J. van Bakel, Foeke J. H. Nauta, Ettore Lanzarone, Frans L. Moll, Joost A. van Herwaarden, Ferdinando Auricchio, Santi Trimarchi

The second affiliation for the 10^th^ author is not indicated. Santi Trimarchi is additionally affiliated with #5 Department of Scienze Biomediche per la Salute, University of Milan, Italy.
